# Genotoxicity Evaluation of Titanium Dioxide Nanoparticles In Vivo and In Vitro: A Meta-Analysis

**DOI:** 10.3390/toxics11110882

**Published:** 2023-10-27

**Authors:** Yue Cao, Jinyao Chen, Qian Bian, Junyu Ning, Ling Yong, Tong Ou, Yan Song, Sheng Wei

**Affiliations:** 1 Key Laboratory of Food Safety Risk Assessment, National Health Commission of the People’s Republic of China (China National Center for Food Safety Risk Assessment), Guangqu Road, Beijing 100022, China; m202175501@hust.edu.cn (Y.C.); yongling@cfsa.net.cn (L.Y.); outong@cfsa.net.cn (T.O.); 2 School of Public Health, Tongji Medical College, Huazhong University of Science and Technology, Hangkong Road, Wuhan 430030, China; 3 Department of Nutrition, Food Safety and Toxicology, West China School of Public Health, Sichuan University, Yihuan Road, Chengdu 610041, China; umbrellayy@163.com; 4 Institute of Toxicology and Risk Assessment, Jiangsu Provincial Center for Disease Control and Prevention, Jiangsu Road, Nanjing 210009, China; bianqian@jscdc.cn; 5 Institute of Toxicology, Beijing Center for Disease Prevention and Control, Hepingli Middle Street, Beijing 100013, China; ningjy@gmail.com

**Keywords:** titanium dioxide, nanoparticles, genotoxicity, hazard evaluation, meta-analysis

## Abstract

Background: Recent studies have raised concerns about genotoxic effects associated with titanium dioxide nanoparticles (TiO_2_ NPs), which are commonly used. This meta-analysis aims to investigate the potential genotoxicity of TiO_2_ NPs and explore influencing factors. Methods: This study systematically searched Chinese and English literature. The literature underwent quality evaluation, including reliability evaluation using the toxicological data reliability assessment method and relevance evaluation using routine evaluation forms. Meta-analysis and subgroup analyses were performed using R software, with the standardized mean difference (SMD) as the combined effect value. Results: A total of 26 studies met the inclusion criteria and passed the quality assessment. Meta-analysis results indicated that the SMD for each genotoxic endpoint was greater than 0. This finding implies a significant association between TiO_2_ NP treatment and DNA damage and chromosome damage both in vivo and in vitro and gene mutation in vitro. Subgroup analysis revealed that short-term exposure to TiO_2_ NPs increased DNA damage. Rats and cancer cells exhibited heightened susceptibility to DNA damage triggered by TiO_2_ NPs (*p* < 0.05). Conclusions: TiO_2_ NPs could induce genotoxicity, including DNA damage, chromosomal damage, and in vitro gene mutations. The mechanism of DNA damage response plays a key role in the genotoxicity induced by TiO_2_ NPs.

## 1. Introduction

Titanium dioxide nanoparticles (TiO_2_ NPs) are particles ranging in size from 1 to 100 nm in at least one dimension of three-dimensional space [1]. Compared to coarse TiO_2_ particles, TiO_2_ NPs exhibit enhanced conductivity, reactivity, photocatalytic activity, and permeability. These outstanding properties have positioned TiO_2_ NPs as one of the most extensively used nanomaterials, finding applications in various industries such as cosmetics, toothpaste, and drug carriers [2,3]. They are also widely used as food additives, primarily added to the coatings of dairy and confectionery products [4].

The unique physicochemical properties of nanoparticles bring about both application advantages and safety concerns. One major concern is that the NPs may increase cellular uptake rate and internalization behavior due to their diminutive size and extensive surface area [5]. Once inside a cell, NPs can disrupt normal cellular functions, leading to cell damage [6]. Moreover, nanomaterials have the potential to interact with molecules within organisms, interfering with biochemical reactions and signaling pathways, thereby affecting the entire biological system [7,8]. Additionally, solubility and ionization play important roles in the cellular responses and toxicity induced by NPs at the molecular, cellular, tissue, and systemic levels [9,10]. Therefore, the risk of adverse effects from TiO_2_ NPs may be amplified when there are additional routes of exposure and high levels of exposure, such as prolonged dermal contact and inhalation.

Evidence suggests TiO_2_ NPs may be genotoxic, including DNA and chromosomal damage. Based on the risk assessment report in 2021, the European Food Safety Authority (EFSA) updated its opinion that food-grade titanium dioxide (E171) was no longer a safe food additive. New research indicated that up to fifty percent of the NPs in E171 could induce DNA strand breakage and chromosome damage [11]. A meta-analysis focusing on the in vitro genotoxicity of TiO_2_ NPs revealed significant increases in tail DNA percentage, olive tail moment, and gene mutation rates [12]. Furthermore, Shi et al. [13] conducted a comprehensive review encompassing both in vivo and in vitro studies on TiO_2_ NPs, which collectively suggested the potential of these NPs to induce genotoxic effects. Both in vivo and in vitro tests confirmed the genotoxic nature of TiO_2_ NPs, with gene mutation and DNA strand breakage serving as sensitive genetic indicators [14]. Notably, the manifestation of genotoxicity depended not only on the particle surface, size, and exposure pathway but also on the duration and concentration of exposure [15].

This study systematically retrieved the latest literature from Chinese and English databases to evaluate the genotoxic effects of TiO_2_ NPs in vivo and in vitro; the selection of the eligible literature adhered to predefined inclusion and exclusion criteria. In addition, a comprehensive quality evaluation of the included literature was conducted, including reliability evaluation based on the toxicological data reliability assessment method and relevance evaluation using routine evaluation forms. The meta-analysis was performed separately for different genotoxic endpoints. Subgroup analyses were used to investigate potential influencing factors, such as particle size, experimental subjects, exposure duration, and exposure concentration. The primary objective of this study was to provide an up-to-date and comprehensive reference for assessing TiO_2_ NPs’ genotoxicity.

## 2. Materials and Methods

### 2.1. Search Strategy

This study comprehensively scoured relevant articles from databases, including PubMed, Web of Science (WoS), China National Knowledge Infrastructure (CNKI), and EFSA reports. The search was conducted using a set of keywords, which included “TiO_2_”, “Titanium dioxide”, “TiO_2_ NPs”, “genotoxicity”, “genotoxic”, “gene”, “DNA”, “chromosome”, and “mutation”. EFSA’s 2016 report on evaluating titanium dioxide as a food additive and common terminology found in CNKI’s translation assistant influenced the choice of these keywords. All papers in English and Chinese published before 30 June 2022 were considered for inclusion in this study.

### 2.2. Selection Criteria

The inclusion criteria in the systematic retrieval included (1) experimental research; (2) studies published in either Chinese or English; (3) mammalian cells or mammals as experimental subjects; (4) studies focused on genotoxic effects, such as gene mutation, chromosome aberration, DNA damage, oxidative stress, etc; and (5) the genotoxicity endpoints reported in the study that included the percentage of DNA in tail (T DNA%), tail length (TL), olive tail moment (OTM), mutation frequency (MF), frequency of micronucleus (MN), percentage of chromosomal aberrations (CA), etc.

The exclusion criteria were also established, including (1) non-original research such as case reports, comments, editorials, reviews, letters, or reports; (2) studies on the joint exposure of TiO_2_ with other substances or ultraviolet rays; (3) studies performed with TiO_2_ nanofibers, nanocomposites, nanotubes, or non-nanoparticles;(4) in vivo studies on non-oral exposure; (5) only epigenetics of genotoxic effects; and (6) no quantitative results or incomplete data.

### 2.3. Quality Assessment

Based on the reliability definition of Klimisch, the toxicological data reliability assessment method (TRAM) was developed to evaluate the reliability of toxicological data. The evaluation process incorporated the meticulous assessment of the physicochemical properties of the substances, as well as the conformance to established design standards governing toxicity tests. TRAM was an ideal tool for undertaking safety assessment in China, as it considered the soundness and validity of the research methodology employed in the studies under review [16].

The TRAM evaluation team comprised 18 experts from Jiangsu’s Center for Disease Control and Prevention (CDC). All members were required to possess professional qualifications in the field of toxicology, including a Master’s degree or higher, a minimum of three years of work experience, and intermediate or senior professional titles. Following TRAM training, the experts evaluated studies based on specific evaluation criteria tailored to different types of data. Each criterion was assigned a weighted score, which was then aggregated and converted into a percentage. Studies that received a score below 60% were categorized as having low reliability, those scoring between 60% and 80% were deemed to have medium reliability, and those scoring above 80% were classified as having high reliability. Studies falling into the “low reliability” category were promptly excluded from subsequent analyses to uphold the analytical rigor and integrity of the process.

Routine evaluation forms were used to determine the relevance of toxicological data. Furthermore, 17 experts from the CDC in Beijing were invited to participate in the evaluation process. The outcome of the evaluation was systematically categorized into three distinct classifications, namely “A”, “B”, or “C”, based on the extent of alignment with the research objectives and the applicability for hazard assessment. Notably, data assigned the label “A” signified a robust concurrence with the research objectives, rendering it highly recommended for hazard evaluation. In contrast, data allocated to the designation “B” embodied a moderated degree of correlation and held the potential for inclusion within the assessment framework. Under circumstances where there was a lack of substantial correlation, the corresponding research was assigned the classification of “C” and consequently excised from any further consideration. Furthermore, to indicate the degree of relevance between exposure route, duration, concentration, and risk assessment, a “+” symbol was added to the results.

### 2.4. Data Extraction

To extract useful information, researchers independently collected and recorded the following contents including (1) basic information, such as the lead author, publication year and country; (2) subject characteristics and interventions, such as species or cells, routes of administration, particle characteristics, treatment time, concentration and sample size (n); and (3) outcome measures, consisting of (a) T DNA%, TL, and OTL in comet assay, (b) MF in gene mutation assay, (c) MN frequency in MN assay, and (d) CA frequency in CA assay. The mean ± standard deviation (SD) was used to describe the outcome variables.

### 2.5. Statistical Analysis

In assessing the combined genotoxic effects of TiO_2_ NPs, the standardized mean difference (SMD) and its 95% confidence interval (CI) were employed. An SMD greater than 0 indicated higher genotoxicity in the exposed groups compared to control groups, while an SMD of 0 suggested no difference between the two groups.

Among the included studies, statistical heterogeneity was estimated by *I*-squared (I^2^) analysis. The significance of heterogeneity was determined by I^2^ > 50 or *p* < 0.05 in the Q-test. In instances where substantial heterogeneity was present among the individual studies, a random-effects model was employed. Conversely, a fixed-effects model was selected for the meta-analysis. Subgroup analysis was performed to identify the potential sources of heterogeneity and to examine the association between treatment variables (e.g., particle size, treatment object, exposure time, and concentration) and the genotoxic effects of TiO_2_ NPs. The stability and reliability of the meta-analysis results were assessed through sensitivity analysis. Considering the limitation of the included literature, a threshold of at least nine studies was established for conducting funnel plots and Egger test analyses to examine the potential for publication bias. All tests were two-tailed, and a significance level of *p* < 0.05 was adopted. R-4.2.0 software and the meta package were utilized for all statistical analyses.

## 3. Results

### 3.1. Literature Screening

**Figure 1:**
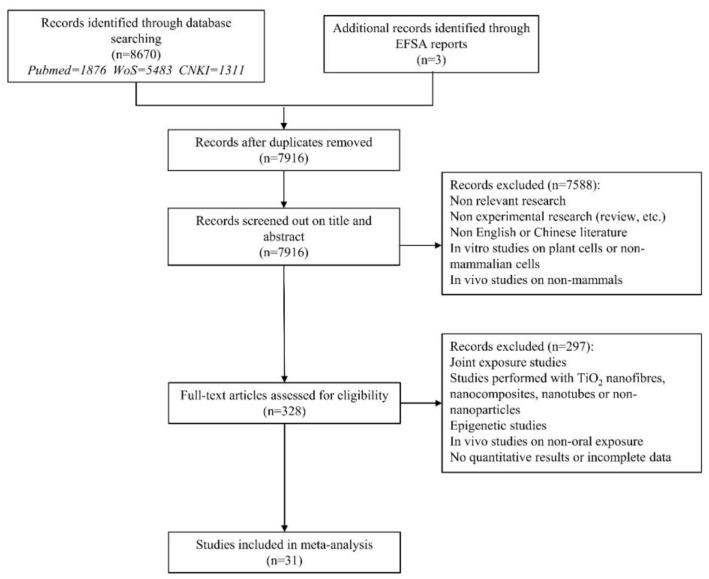
Flow diagram of the literature search and screening.

The process of the literature retrieval and screening is depicted in Figure 1. Of the total retrieved articles, 1876 were obtained from PubMed, 5483 from WoS, and 1311 from CNKI, resulting in a cumulative count of 8670 articles. After excluding 944 duplicate studies, the titles and abstracts of the remaining 7916 records were screened. From this initial filtering, 328 articles were retained for further consideration. Finally, a full-text screening identified 31 studies that met the eligibility criteria for inclusion. Among these, 12 studies were conducted in vivo, while the remaining 19 were conducted in vitro. The focal point of these studies was to discern the genotoxic potential of TiO_2_ NPs.

### 3.2. Basic Characteristics and Quality Assessment

Information from in vivo genotoxicity research of TiO_2_ NPs is summarized in Table 1. The included studies were classified based on outcome indicators as T DNA% (six studies), TL/μm (three studies), OTM/μm (six studies), MN frequency (two studies), and CA frequency (five studies). The results of quality assessments indicated medium to high reliability, with relevance ratings of “B++” and above.

Table 2 provides information on in vitro genotoxicity studies of TiO_2_ NPs. An article with a determined reliability assessment of “low” and four articles exhibiting a correlation evaluation result of “C” were excluded from the meta-analysis. Outcome indicators classified the included studies as T DNA% (nine studies), TL/μm (two studies), OTM/μm (six studies), MF (three studies), MN frequency (seven studies), and CA frequency (two studies).

### 3.3. Meta-Analysis for In Vivo Genotoxicity of TiO_2_ NPs

#### 3.3.1. Heterogeneity Test and Meta-Analysis

The results of the I^2^ analysis for different genotoxic endpoints showed significant heterogeneity (*p* < 0.01, I^2^ ≥ 50%). Consequently, the random-effects model was employed to estimate the combined effects.

**Figure 2:**
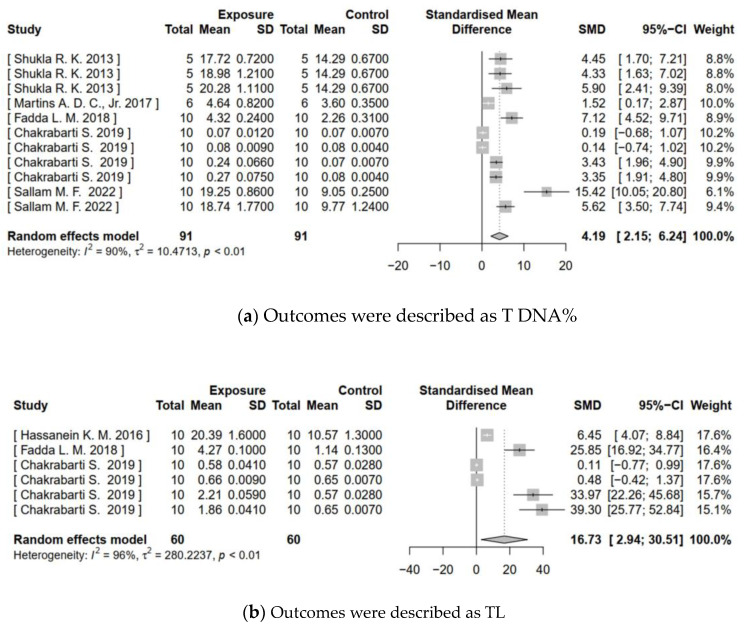

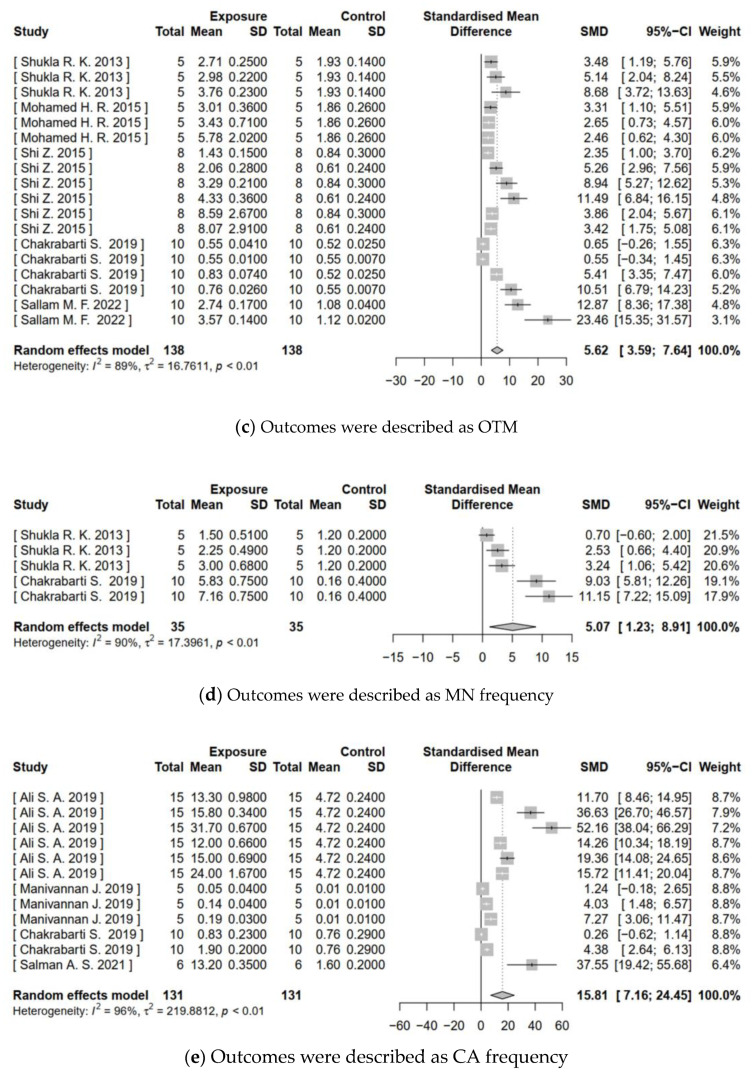
Meta-analysis for in vivo genotoxicity of TiO_2_ NPs. (**a**–**e**) Show the forest plots for genotoxicity endpoints of T DNA%, TL, OTM, MN frequency, and CA frequency, respectively. ‘Total’ is the sample size; ‘SD’ is the standard deviation; ‘SMD’ is the standardized mean difference; ‘95%CI’ is the 95% confidence interval; ‘*I*^2′^ is Higgins’s inconsistency statistic; ‘τ^2′^ is the estimate of between-study variance. Significance is at *p* < 0.05.

Meta-analysis of in vivo genotoxicity of TiO_2_ NPs summarized the SMDs of five categories of genotoxicity endpoints (as shown in Figure 2). The forest plots illustrated significant increases in T DNA% (Z = 4.02, *p* < 0.0001), TL (Z = 2.38, *p* = 0.0174), and OTM (Z = 5.44, *p* < 0.0001). The SMDs and 95%CIs were 4.19 (2.15–6.24), 16.73 (2.94–30.51), and 5.62 (3.59–7.64), respectively, indicating that treatment with TiO_2_ NPs could cause DNA damage. Similarly, MN frequency (Z = 2.59, *p* = 0.0097) and CA frequency (Z = 3.58, *p* = 0.0003) in the exposed group also significantly increased, with SMDs and 95%CIs of 5.07 (1.23–8.91) and 15.81 (7.16–24.45). This evidence suggested that TiO_2_ NPs may induce chromosome damage.

#### 3.3.2. Subgroup Analysis

**Figure 3:**
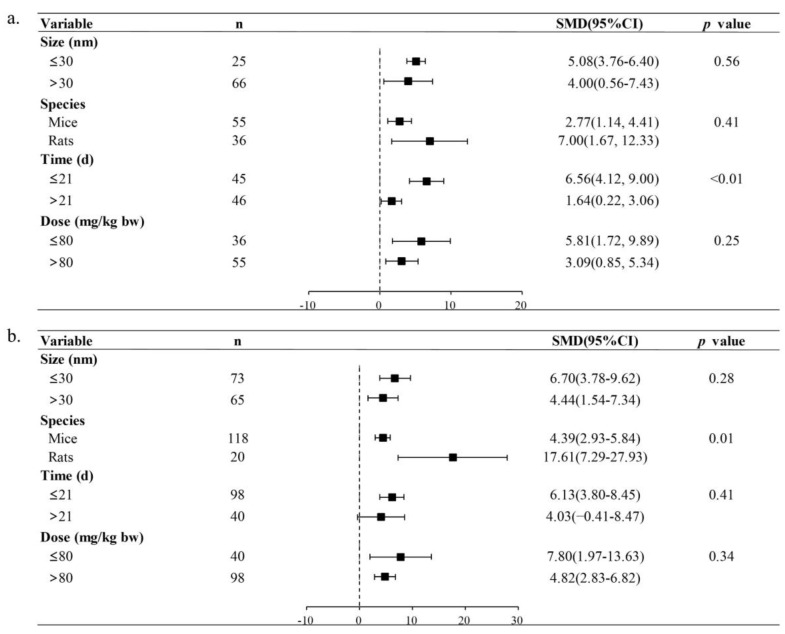
Subgroup analyses of TiO_2_ NPs genotoxicity on in vivo T DNA% (**a**) and OTM (**b**). ’n’ is the sample size; ’SMD’ is the standardized mean difference; ’95%CI’ is the 95% confidence interval; and ‘*p* value’ represents the heterogeneity between subgroups. Significant heterogeneity between subgroups is at *p* < 0.05.

Given the limited available literature on the in vivo genotoxicity of TiO_2_ NPs, our subgroup analysis focused on T DNA% and OTM data. Figure 3 depicts that the observed heterogeneity in the results may be attributed to the exposure time (*p* < 0.01) and the species used in experiments (*p* = 0.01). Specifically, the TiO_2_ NPs-treated group exhibited significantly higher T DNA% in short-term exposures (≤ 21 days) (SMD = 6.56, 95%CI: 4.12–9.00) compared to long-term exposures (>21 days) (SMD = 1.64, 95%CI: 0.22–3.06). Additionally, OTM was significantly higher in rats (SMD = 17.61, 95%CI: 7.29–27.93) than in mice (SMD = 4.39, 95%CI: 2.93–5.84). However, no statistically significant results were observed when considering particle size and treatment dose for T DNA% or OTM. These findings suggested that short-term exposure could potentially contribute to in vivo DNA damage caused by TiO_2_ NPs. Furthermore, rats seem more sensitive to the genotoxic impacts of TiO_2_ NP-induced DNA damage than mice.

#### 3.3.3. Sensitivity Analysis and Publication Bias

The presence of heterogeneity among in vivo studies focusing on various genotoxic endpoints was noted. This analysis did not reveal any significant differences in the study outcomes, as indicated by the SMD and its 95%CI. Considering the relatively limited number of included studies for each genotoxicity endpoint, no publication bias test was performed.

### 3.4. Meta-Analysis for In Vitro Genotoxicity of TiO_2_ NPs

#### 3.4.1. Heterogeneity Test and Meta-Analysis

Due to the observed heterogeneity among in vitro studies with outcome indicators of T DNA%, OTM, and MN frequency (*p* < 0.01), the random-effects model was utilized to analyze the combined effects. Conversely, for the outcome indicators of TL, MF, and CA frequency, which showed no significant heterogeneity, the fixed-effects model was considered appropriate.

**Figure 4:**
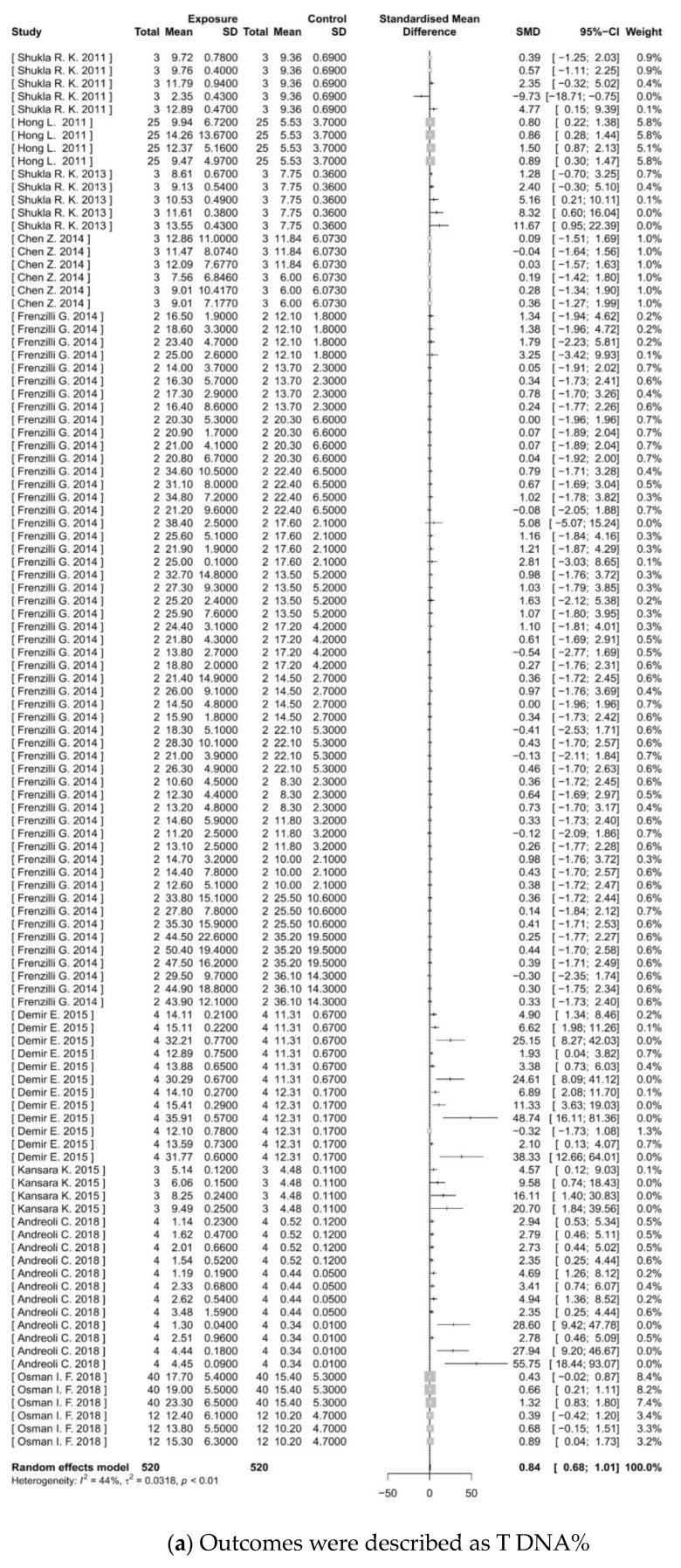

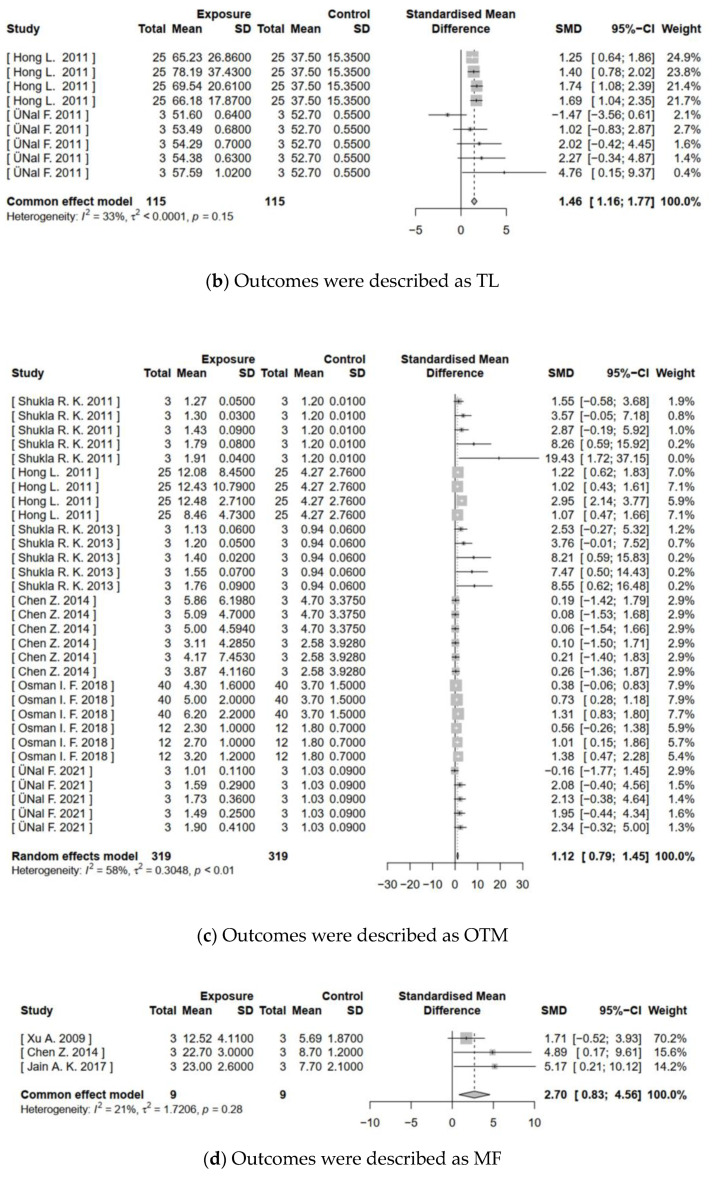

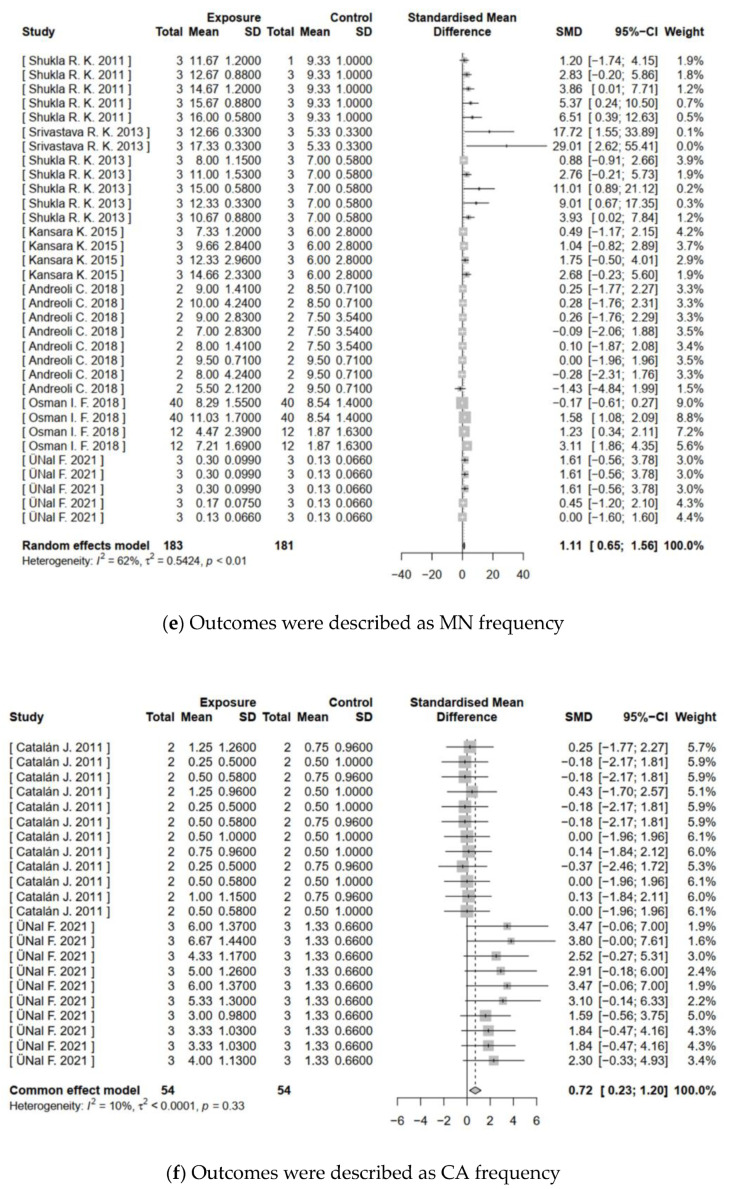
Meta-analysis for in vitro genotoxicity of TiO_2_ NPs. (**a**–**f**) Show the forest plots for genotoxicity endpoints of T DNA%, TL, OTM, MF, MN frequency, and CA frequency, respectively. ‘SD’ is the standard deviation; ‘SMD’ is the standardized mean difference; ‘95%CI’ is the 95% confidence interval; ‘*I*^2′^ is Higgins’s inconsistency statistic; and ‘τ^2′^ is the estimate of between-study variance. Significance is at *p* < 0.05.

The meta-analysis of in vitro genotoxicity of TiO_2_ NPs revealed significant findings across six categories of outcome indicators (as shown in Figure 4). The results from the forest plots indicate that the experimental group exposed to TiO_2_ NPs has significantly higher levels of T DNA% (Z = 10.12, *p* < 0.0001), TL (Z = 9.42, *p* < 0.0001), and OTM (Z = 7.09, *p* < 0.0001) than controls. The SMDs and 95%CIs were 0.84 (0.68–1.01), 1.46 (1.16–1.77), and 1.12 (0.79–1.45), respectively. These findings suggested that TiO_2_ NP treatment could cause DNA damage. There was a significant increase in MF (Z = 2.83, *p* = 0.0046) with a result of 2.70 (0.83–4.56), indicating the potential of TiO_2_ NPs to induce gene mutations. Moreover, significant increases were observed in MN frequency (Z = 5.68, *p* < 0.0001) and CA frequency (Z = 2.90, *p* = 0.0037). The SMDs and 95%CIs were 1.11 (0.65–1.56) and 0.72 (0.23–1.20), respectively, suggesting chromosomal damage effects.

#### 3.4.2. Subgroup Analysis

**Figure 5:**
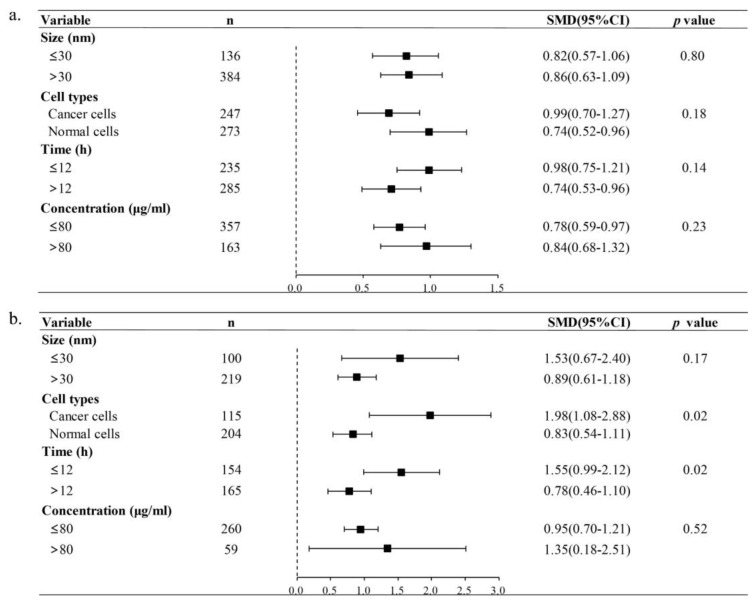
Subgroup analyses of TiO_2_-NPs genotoxicity on in vitro T DNA% (**a**) and OTM (**b**). ’n’ is the sample size; ’SMD’ is the standardized mean difference; ’95%CI’ is the 95% confidence interval; and ‘*p* value’ represents heterogeneity between subgroups. Significant heterogeneity between subgroups is at *p* < 0.05.

In the subgroup analysis conducted on in vitro studies, a specific focus was placed on the examination of T DNA% and OTM data. As illustrated in Figure 5, the potential origins of heterogeneity were identified as the exposure time and the type of experimental cells (*p* = 0.02). For the TiO_2_ NPs-treated group, the results of subgroup analysis revealed that OTM was significantly higher during short-term exposure (≤12 h) (SMD = 1.55, 95%CI: 0.99–2.12) compared to long-term exposure (>12 h) (SMD = 0.78, 95%CI: 0.46–1.10). Furthermore, the OTM value for cancer cells (SMD = 1.98, 95%CI: 1.08–2.88) was significantly higher than that of normal cells (SMD = 0.83, 95%CI: 0.54–1.11). Nevertheless, neither particle size nor exposure concentration exhibited statistically significant differences in relation to T DNA% and OTM. In summation, brief periods of exposure to TiO_2_ NPs may potentially result in DNA damage in vitro. Additionally, cancer cells were discerned to manifest a heightened sensitivity to in vitro DNA damage elicited by TiO_2_ NPs.

#### 3.4.3. Sensitivity Analysis and Publication Bias

The sensitivity analysis of the data from the in vitro assay indicated that no single study significantly impacted the overall results. Furthermore, the merged effect values remained consistent, suggesting that the original results of forest plots were statistically reliable and robust.

**Figure 6:**
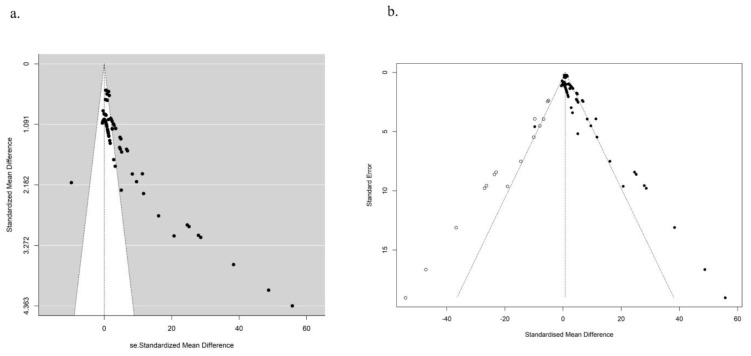
Egger funnel diagram of in vitro T DNA% before (**a**) and after (**b**) publication bias correction by the trim and fill method. The middle line shows the overall estimated standard mean difference. Black dots represent the original studies included, and white dots indicate studies that need supplementation.

A publication bias test was performed specifically for the studies with a genotoxicity endpoint of T DNA%. The funnel plot in Figure 6a revealed an uneven distribution of points representing the effect values for each study. A significant proportion of these points were positioned to the right of the combined effect value and lay outside the associated confidence interval. In addition, the *p*-value of the Egger test was found to be less than 0.05. These findings collectively suggested the presence of publication bias, which possibly affected the accuracy of meta-analysis. To eliminate publication bias, an additional 15 studies were needed, as indicated by hollow origin in Figure 6b.

## 4. Discussion

In this paper, a comprehensive analysis of 12 in vivo and 14 in vitro studies was conducted to assess the genotoxic effects of TiO_2_ NPs. These studies were selected based on meeting the reliability and relevance assessment criteria. The meta-analysis results showed that the SMD for each genotoxic endpoint was greater than 0, suggesting that TiO_2_ NPs significantly induced DNA damage and chromosome damage both in vivo and in vitro. Furthermore, there was a significant association between TiO_2_ NP treatment and gene mutation in vitro. These findings confirmed the potential risks of genotoxicity associated with human exposure to TiO_2_ NPs. Evidently, the duration of exposure and experimental subjects emerged as significant variables influencing DNA damage in the TiO_2_ NPs-treated group. Short-term exposure to TiO_2_ NPs displayed a higher likelihood of inducing DNA damage. The in vivo comet assay revealed that rats exhibited greater sensitivity to DNA damage induced by TiO_2_ NPs than mice. Furthermore, the in vitro comet assay demonstrated that cancer cells exhibited heightened susceptibility to DNA damage induced by TiO_2_ NPs than normal cells. However, it was essential to be cautious about the potential influence of publication bias on the accuracy of the meta-analysis results. 

Currently, three mechanisms have been proposed for the genotoxicity of TiO_2_ NPs. The first mechanism involves direct interaction with DNA. The second one refers to an indirect mechanism in which TiO_2_ NPs interact with other molecules and affect the genetic material. Finally, reactive oxygen species (ROS) are generated due to the catalytic potential of the particles [46]. However, the available evidence questions the direct effect of TiO_2_ NPs on DNA and favors the role of the latter two mechanisms. According to the French Agency for Food, Environmental, and Occupational Health and Safety, there was no evidence of direct interaction between TiO_2_ NPs and DNA or the mitotic apparatus. However, they suggested that direct effects on molecules interacting with genetic material could not be completely excluded [47]. A comprehensive weight of evidence assessment suggested that observed genotoxic effects of TiO_2_ (nano and other forms) were secondary to physiological stress rather than direct DNA damage [48]. Nanoparticle-induced oxidative stress was viewed as a signal transducer for further physiological effects, including genotoxicity and cytotoxicity [49,50]. EFSA concluded that the relative contribution of different molecular mechanisms triggered by TiO_2_ NPs remained unknown [11].

Extensive research has demonstrated that TiO_2_ NP exposure is associated with increased occurrence of DNA damage. This propensity for DNA damage appears to be particularly pronounced following short-term exposure to TiO_2_ NPs. This conclusion is substantiated by the collective findings of all in vivo comet assays and the majority of in vitro comet assays encompassed within this meta-analysis. This aligned with the findings of Ling et al. [12], who also observed severe DNA damage following brief exposure to TiO_2_ NPs. This phenomenon can likely be attributed to the insufficient time for effective DNA repair due to the constricted exposure window. Additionally, comet assay studies showed a correlation between longer exposure periods and reduced DNA damage [51,52]. This implied that TiO_2_ NPs possibly cause early and reversible DNA damage, but cells adapt to the TiO_2_ NPs environment and initiate repair mechanisms during prolonged exposures. The potential impact of genotoxicity includes influencing cellular responses like DNA repair, cell cycle arrest, and apoptosis. Inadequate DNA repair before or during damaged DNA replication could potentially trigger mutagenic and oncogenic events [53].

In comet assay, rats and cancer cells subjected to TiO_2_ NP exposure exhibited a pronounced susceptibility to DNA damage, as evidenced by their significantly higher OTM than mice and normal cells. This observed discrepancy most likely depended on the inherent capacity of DNA damage response (DDR). Cancer cells showed a broad spectrum of mutations and abnormal gene expressions within the domain of DNA repair responses, which set in motion a state of genome instability [54,55]. The frequent compromise of certain DDR pathways in cancer cells facilitated the accumulation of genomic instability. As a result, the loss of functional DDR pathways rendered cancer cells more prone to DNA damage and additional defects within the DDR network [56]. Conversely, the meticulously controlled replication observed in normal cells acted as a buffer against the onset of a hyperactivated DDR [57]. This observation was validated by evidence that the incidence of DNA lesions within cancer cell lines was elevated compared to primary cells cultivated under controlled laboratory conditions [58]. Close attention must be paid to the risks of cancer treatments based on TiO_2_ NP drug delivery systems [59].Studies have shown that exposure to TiO_2_ NPs of high concentrations or small size is usually associated with higher genotoxicity. A literature review concluded that genotoxicity exhibited an increasing trend with decreasing particle size and increasing concentrations of TiO_2_ NPs [13]. Moreover, Dubey et al. [60] observed a dose-dependent escalation in DNA damage, lipid peroxidation, and protein carbonylation as concentrations of exposed nanoparticles increased. In this study, no difference in DNA damage induced by TiO_2_ NPs was observed under varying particle sizes and exposure concentrations. More high-quality literature is needed to be included in the comprehensive analysis.

The impact of TiO_2_ NPs on gene mutation and chromosome aberrations has been extensively studied. Jain et al. [43] reported a linear correlation between the mutation rates and the exposure levels of TiO_2_ NPs. Moreover, the mutagenic potential of TiO_2_ NPs in V-79 cells was evaluated via mammalian HGPRT gene forward mutation assay, showing a 2.98-fold increase in 6TG^R^ HGPRT mutant frequency [42]. The presence of heightened levels of ROS could interact with cellular components, including DNA bases or the deoxyribosyl backbone of DNA, resulting in the formation of damaged bases or strand breaks. Certain oxidative DNA lesions, which might not be fully repaired, could act as precursors to mutagenesis. This phenomenon is particularly relevant to mismatch repair or incomplete repair mechanisms, which can give rise to specific mutational events [42,61]. The study employing transmission electron microscopy yielded evidence suggesting that the internalization of TiO_2_ NPs by cells is observable within cytoplasmic vesicles and close to and inside the nucleus. Notably, larger agglomerates of TiO_2_ NPs were believed to possess the capacity to disrupt or damage chromosomal structures, potentially leading to chromosome aberrations [62]. This meta-analysis incorporated the most recent studies of in vivo and in vitro genotoxicity and underwent rigorous quality assessments to enable quantitative analysis. However, there were still some limitations. The available data were primarily limited as only the Chinese and English literature was included in the screening process. However, the high reliability and relevance of the included literature increased confidence in the results. Secondly, it is suggested that future studies pay closer attention to the substance characterization of TiO_2_ NPs, such as shape, size, and charge. The association between these important characteristics and genotoxicity is worth discussing in depth. Finally, more high-quality genotoxicity studies on TiO_2_ NPs are needed to help minimize the impact of publication bias.

Moving forward, there are several key aspects that researchers should focus on in future studies concerning TiO_2_ NPs and genotoxicity. Long-term animal studies would be valuable to explore the underlying molecular mechanisms of genotoxicity induced by TiO_2_ NPs further. Researchers should also investigate the catabolism of TiO_2_ NPs once they enter the human body. Study results will provide valuable insights into the internal exposure dose of nanoparticles within target organs or cells. Furthermore, establishing a cut-off value for TiO_2_ particle size in relation to genotoxicity is an important area of research. Establishing stringent regulations and guidelines for the judicious application of TiO_2_ NPs is essential to mitigate their potential genotoxic effects, thus ensuring effective protection of public health.

## 5. Conclusions

This meta-analysis has provided evidence that TiO_2_ NPs could induce genotoxicity, including DNA damage and chromosomal damage both in vivo and in vitro, as well as in vitro gene mutations. Short-term exposure to TiO_2_ NPs would lead to increased DNA damage. Rats were more sensitive to TiO_2_ NPs-induced DNA damage in vivo than mice, and cancer cells exhibited heightened susceptibility to in vitro DNA damage induced by TiO_2_ NPs than normal cells. The interaction between TiO_2_ NPs and DNA, along with the activation of ROS, influenced the DNA repair response and induced genotoxicity. Therefore, it is necessary to raise public awareness about the potential risks associated with using TiO_2_ NPs, particularly in products intended for consumption as food and drugs.

## Figures and Tables

**Table 1:** Basic characteristics and quality evaluation of the included studies on in vivo genotoxicity of TiO_2_ NPs ^1^.

Included Studies	Country	Test Animals and Exposure Methods	TiO_2_-NP Characteristics			Dose (mg/kg bw)	Exposure		Control		Reliability Evaluation	Correlation Evaluation
			Crystal	Size (nm)	Purity (%)		*n*	Mean ± SD	*n*	Mean ± SD		
Outcomes were described as T DNA%												
Shukla R. K. 2014 [17]	India	*Male Swiss albino mice* (continuous gavage for 14 d)	Anatase	20–50	99.7	10	5	17.72 ± 0.72	5	14.29 ± 0.67	high	A+++
						50	5	18.98 ± 1.21	5	14.29 ± 0.67		
						100	5	20.28 ± 1.11	5	14.29 ± 0.67		
Martins A. D. C., Jr. 2017 [18]	Brazil	*Male Wistar rats* (continuous gavage for 45 d)	NA	41.99 ± 1.63	NA	0.5	6	4.64 ± 0.82	6	3.6 ± 0.35	medium	B+++
Fadda L. M. 2018 [19]	Saudi Arabia	*Male Wistar Albino rats* (continuous gavage for 21 d)	Anatase	60 ± 10	NA	1000	10	4.32 ± 0.24	10	2.26 ± 0.31	medium	B+++
Chakrabarti S. 2019 [20]	India	*Female/male Swiss-Albino mice* (oral for 90 d)	NA	58.25 ± 8.11	NA	200	10	0.07 ± 0.012 (liver) 0.085 ± 0.009 (kidney)	10	0.068 ± 0.007 (liver) 0.084 ± 0.004 (kidney)	high	A+++
						500	10	0.236 ± 0.066 (liver) 0.27 ± 0.075 (kidney)	10	0.068 ± 0.007 (liver) 0.084 ± 0.004 (kidney)		
Sallam M. F. 2022 [21]	Egypt	*Male SD rats* (continuous gavage for 21 d)	NA	50 ± 2.4	NA	50	10	19.25 ± 0.86	10	9.05 ± 0.25	medium	B++
Sallam M. F. 2022 [22]	Egypt	*Male SD rats* (continuous gavage for 21 d)	NA	28	NA	50	10	18.74 ± 1.77	10	9.77 ± 1.24	medium	B++
Outcomes were described as TL (μm)												
Hassanein K. M. 2016 [23]	Egypt	*Adult* male *SD rats* (continuous gavage for 90 d)	NA	21	NA	150	10	20.39 ± 1.6	10	10.57 ± 1.3	medium	A+++
Fadda L. M. 2018 [19]	Saudi Arabia	*Male Wistar Albino rats* (continuous gavage for 21 d)	NA	60 ± 10	NA	1000	10	4.27 ± 0.10	10	1.14 ± 0.13	medium	B+++
Chakrabarti S. 2019 [20]	India	*Female/male Swiss-Albino mice* (oral for 90 d)	NA	58.25 ± 8.11	NA	200	10	0.579 ± 0.041 (liver) 0.655 ± 0.009 (kidney)	10	0.575 ± 0.028 (liver) 0.651 ± 0.007 (kidney)	high	A+++
						500	10	2.213 ± 0.059 (liver) 1.858 ± 0.041 (kidney)	10	0.575 ± 0.028 (liver) 0.651 ± 0.007 (kidney)		
Outcomes were described as OTM (μm)												
Shukla R. K. 2014 [17]	India	*Male Swiss albino mice* (continuous gavage for 14 d)	Anatase	20–50	99.7	10	5	2.71 ± 0.25	5	1.93 ± 0.14	high	A+++
						50	5	2.98 ± 0.22	5	1.93 ± 0.14		
						100	5	3.76 ± 0.23	5	1.93 ± 0.14		
Mohamed H. R. 2015 [24]	Egypt	*Male Swiss Webster mice* (continuous gavage for 5 d)	Anatase/Rutile	46.23 ± 3.45	99.5	5	5	3.01 ± 0.36	5	1.86 ± 0.26	medium	B+++
						50	5	3.43 ± 0.71	5	1.86 ± 0.26		
						500	5	5.78 ± 2.02	5	1.86 ± 0.26		
Shi Z. 2015 [25]	China	*Female/male wild-type ICR mice, Nrf2(-/-) ICR mice* (continuous gavage for 7 d)	Anatase	10–25	99.7	500	8	1.43 ± 0.15 (liver) 2.06 ± 0.28 (kidney)	8	0.84 ± 0.30 (liver) 0.61 ± 0.24 (kidney)	high	A+++
						1000	8	3.29 ± 0.21 (liver) 4.33 ± 0.36 (kidney)	8	0.84 ± 0.30 (liver) 0.61 ± 0.24 (kidney)		
						2000	8	8.59 ± 2.67 (liver) 8.07 ± 2.91 (kidney)	8	0.84 ± 0.30 (liver) 0.61 ± 0.24 (kidney)		
Chakrabarti S. 2019 [20]	India	*Female/male Swiss-Albino mice* (oral for 90 d)	NA	58.25 ± 8.11	NA	200	10	0.546 ± 0.041 (liver) 0.554 ± 0.01 (kidney)	10	0.523 ± 0.025 (liver) 0.549 ± 0.007 (kidney)	high	A+++
						500	10	0.835 ± 0.074 (liver) 0.758 ± 0.026 (kidney)	10	0.523 ± 0.025 (liver) 0.549 ± 0.007 (kidney)		
Sallam M. F. 2022 [21]	Egypt	*Male SD rats* (continuous gavage for 21 d)	NA	50 ± 2.4	NA	50	10	2.74 ± 0.17	10	1.08 ± 0.04	medium	B++
Sallam M. F. 2022 [22]	Egypt	*Male SD rats* (continuous gavage for 21 d)	NA	28	NA	50	10	3.57 ± 0.14	10	1.12 ± 0.02	medium	B++
Outcomes were described as MN frequency (MN/1000 PCEs)												
Shukla R. K. 2014 [17]	India	*Male Swiss albino mice* (continuous gavage for 14 d)	Anatase	20–50	99.7	10	5	1.50 ± 0.51	5	1.20 ± 0.20	high	A+++
						50	5	2.25 ± 0.49	5	1.20 ± 0.20		
						100	5	3.0 ± 0.68	5	1.20 ± 0.20		
Chakrabarti S. 2019 [20]	India	*Female/male Swiss-Albino mice* (oral for 90 d)	NA	58.25 ± 8.11	NA	200	10	5.83 ± 0.75	10	0.16 ± 0.40	high	A+++
						500	10	7.16 ± 0.75	10	0.16 ± 0.40		
Outcomes were described as CA frequency												
Ali S. A. 2019 [26]	Egypt	*Male Swiss albino mice* (continuous oral for 5 d)	NA	21	NA	50	15	13.30 ± 0.98	15	4.72 ± 0.24	medium	A+++
						250	15	15.80 ± 0.34	15	4.72 ± 0.24		
						500	15	31.70 ± 0.67	15	4.72 ± 0.24		
Ali S. A. 2019 [26]	Egypt	*Male Swiss albino mice* (continuous oral for 5 d)	NA	80	NA	50	15	12.00 ± 0.66	15	4.72 ± 0.24	medium	A+++
						250	15	15.00 ± 0.69	15	4.72 ± 0.24		
						500	15	24.00 ± 1.67	15	4.72 ± 0.24		
Manivannan J. 2019 [27]	India	*Male Swiss albino mice* (continuous gavage for 28 d)	Rutile	25.074 ± 3.593	NA	0.2	5	0.05 ± 0.04	5	0.01 ± 0.01	high	B+++
						0.4	5	0.14 ± 0.04	5	0.01 ± 0.01		
						0.8	5	0.19 ± 0.03	5	0.01 ± 0.01		
Chakrabarti S. 2019 [20]	India	*Female/male Swiss-Albino mice* (oral for 90 d)	NA	58.25 ± 8.11	NA	200	10	0.83 ± 0.23	10	0.76 ± 0.29	high	A+++
						500	10	1.9 ± 0.20	10	0.76 ± 0.29		
Salman A. S. 2021 [28]	Germany	*Male Balb/c mice* (continuous gavage for 21 d)	NA	28.9	NA	25	6	13.2 ± 0.35	6	1.6 ± 0.2	high	A+++

^1^ NA: not applicable; *n*: sample size; SD: standard deviation; T DNA%: the percentage of DNA in tail; TL: tail length; OTM: olive tail moment; MF: mutation frequency; MN/1000 PCEs: no. of micronucleus/1000 polychromatic erythrocytes; CA frequency: percentage of cells exhibiting chromosomal aberrations.

**Table 2:** Basic characteristics and quality evaluation of the included studies on in vitro genotoxicity of TiO_2_ NPs ^1^.

Included Studies	Country	Test Cells and Exposure Methods	TiO_2_-NP Characteristics			Concentration (μg/mL)	Exposure		Control		Reliability Evaluation	Correlation Evaluation
			Crystal	Size (nm)	Purity (%)		*n*	Mean ± SD	*n*	Mean ± SD		
Outcomes were described as T DNA%												
Shukla R. K. 2011 [29]	India	*Human epidermal cells line A431*, exposed for 6 h	Anatase	50	99.7	0.008	3	9.72 ± 0.78	3	9.36 ± 0.69	high	B
						0.08	3	9.76 ± 0.40	3	9.36 ± 0.69		
						0.8	3	11.79 ± 0.94	3	9.36 ± 0.69		
						8	3	2.35 ± 0.43	3	9.36 ± 0.69		
						80	3	12.89 ± 0.47	3	9.36 ± 0.69		
Hong L. 2011 [30]	China	*Human lung adenocarcinoma cells*, exposed for 6 h	NA	5–10	>99.9	25	25	9.94 ± 6.72	25	5.53 ± 3.70	medium	A+++
						50	25	14.26 ± 13.67	25	5.53 ± 3.70		
						100	25	12.37 ± 5.16	25	5.53 ± 3.70		
						200	25	9.47 ± 4.97	25	5.53 ± 3.70		
Shukla R. K. 2013 [31]	India	*HepG2 human hepatocellular carcinoma cells*, exposed for 6 h	Anatase	30–70	99.7	1	3	8.61 ± 0.67	3	7.75 ± 0.36	high	B
						10	3	9.13 ± 0.54	3	7.75 ± 0.36		
						20	3	10.53 ± 0.49	3	7.75 ± 0.36		
						40	3	11.61 ± 0.38	3	7.75 ± 0.36		
						80	3	13.55 ± 0.43	3	7.75 ± 0.36		
Chen Z. 2014 [14]	China	*V79 cells*, exposed for 6 h, 24 h	Anatase	75 ± 15	99.90	5	3	12.863 ± 11.00(6 h) 7.557 ± 6.846(24 h)	3	11.836 ± 6.073(6 h) 6.000 ± 6.866(24 h)	high	A+++
						20	3	11.470 ± 8.074(6 h) 9.007 ± 10.417(24 h)	3	11.836 ± 6.073(6 h) 6.000 ± 6.866(24 h)		
						100	3	12.094 ± 7.677(6 h) 9.005 ± 7.177(24 h)	3	11.836 ± 6.073(6 h) 6.000 ± 6.866(24 h)		
Frenzilli G. 2014 [32]	Italy	*Human fibroblast (HuDE)*, exposed for 4 h, 24 h and 48 h	Anatase	20–50	99.7	20	2	16.5 ± 1.9(4 h) 14.0 ± 3.7(24 h) 20.3 ± 5.3(48 h)	2	12.1 ± 1.8(4 h) 13.7 ± 2.3(24 h) 20.3 ± 6.6(48 h)	medium	B+
						50	2	18.6 ± 3.3(4 h) 16.3 ± 5.7(24 h) 20.9 ± 1.7(48 h)	2	12.1 ± 1.8(4 h) 13.7 ± 2.3(24 h) 20.3 ± 6.6(48 h)		
						100	2	23.4 ± 4.7(4 h) 17.3 ± 2.9(24 h) 21.0 ± 4.1(48 h)	2	12.1 ± 1.8(4 h) 13.7 ± 2.3(24 h) 20.3 ± 6.6(48 h)		
						150	2	25.0 ± 2.6(4 h) 16.4 ± 8.6(24 h) 20.8 ± 6.7(48 h)	2	12.1 ± 1.8(4 h) 13.7 ± 2.3(24 h) 20.3 ± 6.6(48 h)		
Frenzilli G. 2014 [32]	Italy	*Bottlenose dolphin fibroblast (BDF)*, exposed for 4 h, 24 h and 48 h	Anatase	20–50	99.7	20	2	34.6 ± 10.5(4 h) 38.4 ± 2.5(24 h) 32.7 ± 14.8(48 h)	2	22.6 ± 6.5(4 h) 17.6 ± 2.1(24 h) 13.5 ± 5.2(48 h)	medium	B+
						50	2	31.1 ± 8.0(4 h) 25.6 ± 5.1(24 h) 27.3 ± 9.3(48 h)	2	22.6 ± 6.5(4 h) 17.6 ± 2.1(24 h) 13.5 ± 5.2(48 h)		
						100	2	34.8 ± 7.2(4 h) 21.9 ± 1.9(24 h) 25.2 ± 2.4(48 h)	2	22.6 ± 6.5(4 h) 17.6 ± 2.1(24 h) 13.5 ± 5.2(48 h)		
						150	2	21.2 ± 9.6(4 h) 25.0 ± 0.1(24 h) 25.9 ± 7.6(48 h)	2	22.6 ± 6.5(4 h) 17.6 ± 2.1(24 h) 13.5 ± 5.2(48 h)		
Frenzilli G. 2014 [32]	Italy	*Mouse fibroblast (3 T3)*, exposed for 4 h, 24 h and 48 h	Anatase	20–50	99.7	20	2	24.4 ± 3.1(4 h) 21.4 ± 14.9(24 h) 18.3 ± 5.1(48 h)	2	17.2 ± 4.2(4 h) 14.5 ± 2.7(24 h) 22.1 ± 5.3(48 h)	medium	B+
						50	2	21.8 ± 4.3(4 h) 26.0 ± 9.1(24 h) 28.3 ± 10.1(48 h)	2	17.2 ± 4.2(4 h) 14.5 ± 2.7(24 h) 22.1 ± 5.3(48 h)		
						100	2	13.8 ± 2.7(4 h) 14.5 ± 4.8(24 h) 21.0 ± 3.9(48 h)	2	17.2 ± 4.2(4 h) 14.5 ± 2.7(24 h) 22.1 ± 5.3(48 h)		
						150	2	18.8 ± 2.0(4 h) 15.9 ± 1.8(24 h) 26.3 ± 4.9(48 h)	2	17.2 ± 4.2(4 h) 14.5 ± 2.7(24 h) 22.1 ± 5.3(48 h)		
Frenzilli G. 2014 [32]	Italy	*Human leukocytes (HL)*, exposed for 4 h, 24 h and 48 h	Anatase	20–50	99.7	20	2	10.6 ± 4.5(4 h) 14.6 ± 5.9(24 h) 14.7 ± 3.2(48 h)	2	8.3 ± 2.3(4 h) 11.8 ± 3.2(24 h) 10.0 ± 2.1(48 h)	medium	B+
						50	2	12.3 ± 4.4(4 h) 11.2 ± 2.5(24 h) 14.4 ± 7.8(48 h)	2	8.3 ± 2.3(4 h) 11.8 ± 3.2(24 h) 10.0 ± 2.1(48 h)		
						100	2	13.2 ± 4.8(4 h) 13.1 ± 2.5(24 h) 12.6 ± 5.1(48 h)	2	8.3 ± 2.3(4 h) 11.8 ± 3.2(24 h) 10.0 ± 2.1(48 h)		
Frenzilli G. 2014 [32]	Italy	*Bottlenose dolphin leukocytes (BDL)*, exposed for4 h, 24 h and 48 h	Anatase	20–50	99.7	20	2	33.8 ± 15.1(4 h) 44.5 ± 22.6(24 h) 29.5 ± 9.7(48 h)	2	25.5 ± 10.6(4 h) 35.2 ± 19.5(24 h) 36.1 ± 14.3(48 h)	medium	B+
						50	2	27.8 ± 7.8(4 h) 50.4 ± 19.4(24 h) 44.9 ± 18.8(48 h)	2	25.5 ± 10.6(4 h) 35.2 ± 19.5(24 h) 36.1 ± 14.3(48 h)		
						100	2	35.3 ± 15.9(4 h) 47.5 ± 16.2(24 h) 43.9 ± 12.1(48 h)	2	25.5 ± 10.6(4 h) 35.2 ± 19.5(24 h) 36.1 ± 14.3(48 h)		
Demir E. 2015 [33]	Spain	*Human embryonic kidney cells (HEK293)*, cultured for 1 h	Rutile	21	≥99.5	10	4	14.11 ± 0.21	4	11.31 ± 0.67	high	A
						100	4	15.11 ± 0.22	4	11.31 ± 0.67		
						1000	4	32.21 ± 0.77	4	11.31 ± 0.67		
				50	≥98	10	4	12.89 ± 0.75	4	11.31 ± 0.67		
						100	4	13.88 ± 0.65	4	11.31 ± 0.67		
						1000	4	30.29 ± 0.67	4	11.31 ± 0.67		
Demir E. 2015 [33]	Spain	*Mouse embryonic kidney cells (NIH/3 T3)*, cultured for 1 h	Rutile	21	≥99.5	10	4	14.10 ± 0.27	4	12.31 ± 0.17	high	A
						100	4	15.41 ± 0.29	4	12.31 ± 0.17		
						1000	4	35.91 ± 0.57	4	12.31 ± 0.17		
				50	≥98	10	4	12.10 ± 0.78	4	12.31 ± 0.17		
						100	4	13.59 ± 0.73	4	12.31 ± 0.17		
						1000	4	31.77 ± 0.60	4	12.31 ± 0.17		
Kansara K. 2015 [34]	India	*Human lung cancer cell line (A549)*, exposed for 6 h	Rutile	4–8	99.7	25	3	5.14 ± 0.12	3	4.48 ± 0.11	medium	B
						50	3	6.06 ± 0.15	3	4.48 ± 0.11		
						75	3	8.25 ± 0.24	3	4.48 ± 0.11		
						100	3	9.49 ± 0.25	3	4.48 ± 0.11		
Andreoli C. 2018 [35]	Italy	*Peripheral blood monocytes*, exposed for 24 h	Anatase	20–60	>99.5	10	4	1.14 ± 0.23	4	0.52 ± 0.12	medium	A
						50	4	1.62 ± 0.47	4	0.52 ± 0.12		
						100	4	2.01 ± 0.66	4	0.52 ± 0.12		
						200	4	1.54 ± 0.52	4	0.52 ± 0.12		
Andreoli C. 2018 [35]	Italy	*Peripheral blood monocytes*, exposed for 24 h	Rutile	30 × 100	>99.5	10	4	1.19 ± 0.19	4	0.44 ± 0.05	medium	A
						50	4	2.33 ± 0.68	4	0.44 ± 0.05		
						100	4	2.62 ± 0.54	4	0.44 ± 0.05		
						200	4	3.48 ± 1.59	4	0.44 ± 0.05		
Andreoli C. 2018 [35]	Italy	*Peripheral blood monocytes*, exposed for 24 h	Anatase/Rutile	45–262	>99.5	10	4	1.30 ± 0.04	4	0.34 ± 0.01	medium	A
						50	4	2.51 ± 0.96	4	0.34 ± 0.01		
						100	4	4.44 ± 0.18	4	0.34 ± 0.01		
						200	4	4.45 ± 0.09	4	0.34 ± 0.01		
Osman I. F. 2018 [36]	UK	*Lymphocytes from patients with respiratory diseases*, exposed for 72 h	Anatase	40–70	99.7	10	40	17.7 ± 5.4	40	15.4 ± 5.3	high	B
						30	40	19.0 ± 5.5	40	15.4 ± 5.3		
						50	40	23.3 ± 6.5	40	15.4 ± 5.3		
Osman I. F. 2018 [36]	UK	*Lymphocytes from healthy people*, exposed for 72 h	Anatase	40–70	99.7	10	12	12.4 ± 6.1	12	10.2 ± 4.7	high	B
						30	12	13.8 ± 5.5	12	10.2 ± 4.7		
						50	12	15.3 ± 6.3	12	10.2 ± 4.7		
Outcomes were described as TL (μm)												
Hong L. 2011 [30]	China	*Human lung adenocarcinoma cells*, exposed for 6 h	NA	5–10	>99.9	25	25	65.23 ± 26.86	25	37.50 ± 15.35	medium	A+++
						50	25	78.19 ± 37.43	25	37.50 ± 15.35		
						100	25	69.54 ± 20.61	25	37.50 ± 15.35		
						200	25	66.18 ± 17.87	25	37.50 ± 15.35		
Ünal F. 2021 [37]	Turkey	*Human lymphocytes*, exposed for 30 min	NA	<100	NA	20	3	51.60 ± 0.64	3	52.70 ± 0.55	medium	A+++
						40	3	53.49 ± 0.68	3	52.70 ± 0.55		
						60	3	54.29 ± 0.70	3	52.70 ± 0.55		
						80	3	54.38 ± 0.63	3	52.70 ± 0.55		
						100	3	57.59 ± 1.02	3	52.70 ± 0.55		
Outcomes were described as OTM (μm)												
Shi Y. 2010 [38]	China	*Human fetal liver L-02 cells*, exposed for 24 h	Anatase/Rutile	30–50	NA	0.01	9	0.91 ± 0.75	9	0.79 ± 0.74	high	C
						0.1	9	1.28 ± 0.96	9	0.79 ± 0.74		
						1	9	1.30 ± 1.01	9	0.79 ± 0.74		
Du H. 2012 [39]	China	*Human fetal liver L-02 cells*, exposed for 24 h	NA	25–50	>99.5	0.001	3	0.67 ± 0.09	3	0.65 ± 0.06	median	C
						0.01	3	0.68 ± 0.10	3	0.65 ± 0.06		
						0.1	3	0.71 ± 0.08	3	0.65 ± 0.06		
						1	3	0.73 ± 0.09	3	0.65 ± 0.06		
						10	3	0.76 ± 0.09	3	0.65 ± 0.06		
Shukla R. K. 2011 [29]	India	*Human epidermal cell line A431*, exposed for 6 h	Anatase	50	99.7	0.008	3	1.27 ± 0.05	3	1.20 ± 0.01	high	B
						0.08	3	1.30 ± 0.03	3	1.20 ± 0.01		
						0.8	3	1.43 ± 0.09	3	1.20 ± 0.01		
						8	3	1.79 ± 0.08	3	1.20 ± 0.01		
						80	3	1.91 ± 0.04	3	1.20 ± 0.01		
Hong L. 2011 [30]	China	*Human lung adenocarcinoma cells*, exposed for 6 h	NA	5–10	>99.9	25	25	12.08 ± 8.45	25	4.27 ± 2.76	medium	A+++
						50	25	12.43 ± 10.79	25	4.27 ± 2.76		
						100	25	12.48 ± 2.71	25	4.27 ± 2.76		
						200	25	8.46 ± 4.73	25	4.27 ± 2.76		
Shukla R. K. 2013 [31]	India	*HepG2 human hepatocellular hepatoma cells*, exposed for 6 h	Anatase	30–70	99.7	1	3	1.13 ± 0,06	3	0.94 ± 0.06	high	B
						10	3	1.20 ± 0.05	3	0.94 ± 0.06		
						20	3	1.40 ± 0.02	3	0.94 ± 0.06		
						40	3	1.55 ± 0.07	3	0.94 ± 0.06		
						80	3	1.76 ± 0.09	3	0.94 ± 0.06		
Chen Z. 2014 [14]	China	*V79 cells*, exposed for 6 h, 24 h	Anatase	75 ± 15	99.90	5	3	5.857 ± 6.198(6 h) 3.113 ± 4.285(24 h)	3	4.698 ± 3.375(6 h) 2.576 ± 3.928(24 h)	high	A+++
						20	3	5.086 ± 4.700(6 h) 4.174 ± 7.453(24 h)	3	4.698 ± 3.375(6 h) 2.576 ± 3.928(24 h)		
						100	3	4.999 ± 4.594(6 h) 3.870 ± 4.116(24 h)	3	4.698 ± 3.375(6 h) 2.576 ± 3.928(24 h)		
Ryu A. R. 2016 [40]	Korea	*Peripheral blood lymphocytes of rats*, exposed for 30 min	NA	NA	NA	60	6	23.08 ± 0.52	6	8.79 ± 2.18	low	B
						80	6	25.66 ± 6.11	6	8.79 ± 2.18		
Osman I. F. 2018 [36]	UK	*Lymphocytes from patients with respiratory diseases*, exposed for 72 h	Anatase	40–70	99.7	10	40	4.3 ± 1.6	40	3..7 ± 1.5	high	B
						30	40	5.0 ± 2.0	40	3..7 ± 1.5		
						50	40	6.2 ± 2.2	40	3..7 ± 1.5		
Osman I. F. 2018 [36]	UK	*Lymphocytes from healthy people*, exposed for 72 h	Anatase	40–70	99.7	10	12	2.3 ± 1.0	12	1.8 ± 0.7	high	B
						30	12	2.7 ± 1.0	12	1.8 ± 0.7		
						50	12	3.2 ± 1.2	12	1.8 ± 0.7		
Ünal F. 2021 [37]	Turkey	*Human lymphocytes*, exposed for 30 min	NA	<100	NA	20	3	1.01 ± 0.11	3	1.03 ± 0.09	medium	A+++
						40	3	1.59 ± 0.29	3	1.03 ± 0.09		
						60	3	1.73 ± 0.36	3	1.03 ± 0.09		
						80	3	1.49 ± 0.25	3	1.03 ± 0.09		
						100	3	1.90 ± 0.41	3	1.03 ± 0.09		
Outcomes were described as MF												
Xu A. 2009 [41]	US	*Primary embryonic fibroblasts of transgenic mice*, incubated in medium for 24 h	Anatase	5	99.7	0.1	3	12.52 ± 4.11	3	5.69 ± 1.87	medium	B
Chen Z. 2014 [14]	China	*V79 cells*, exposed for 24 h	Anatase	75 ± 15	99.9	100	3	22.7 ± 3.0	3	8.7 ± 1.2	high	A+++
Jain A. K. 2017 [42]	India	*Chinese hamster lung fibroblasts (V-79)*, exposed for 6 h	Anatase	12–25	99.7	100	3	23.0 ± 2.6	3	7.7 ± 2.1	medium	A++
Outcomes were described as MN frequency (BiMN)												
Shi Y. 2010 [38]	China	*Human fetal liver L-02 cells*, exposed for 24 h	Anatase/Rutile	30–50	NA	0.01	9	0.91 ± 0.75	9	0.79 ± 0.74	high	C
						0.1	9	1.28 ± 0.96	9	0.79 ± 0.74		
						1	9	1.30 ± 1.01	9	0.79 ± 0.74		
Kang S. J. 2008 [43]	South Korea	*Peripheral blood lymphocytes*, exposed for 20 h	Anatase/Rutile	25	NA	20	3	15.00 ± 1.00	3	9.33 ± 1.52	median	C
						50	3	18.33 ± 2.08	3	9.33 ± 1.52		
						100	3	23.67 ± 0.58	3	9.33 ± 1.52		
Reis É.deM 2016 [44]	Brazil	*V79 cells*, exposed for 3 h	Anatase	3.4	99.7	30	3	6.67 ± 1.15	3	7.00 ± 1.00	high	C
						60	3	12.00 ± 1.00	3	7.00 ± 1.00		
						120	3	14.67 ± 2.06	3	7.00 ± 1.00		
Reis É.deM 2016 [44]	Brazil	*V79 cells*, exposed for 3 h	Anatase	6.2	99.7	30	3	11.33 ± 2.31	3	7.00 ± 1.00	high	C
						60	3	8.33 ± 1.15	3	7.00 ± 1.00		
						120	3	10.00 ± 2.00	3	7.00 ± 1.00		
Reis É.deM 2016 [44]	Brazil	*V79 cells*, exposed for 3 h	Anatase	78	99.7	30	3	5.33 ± 1.53	3	7.00 ± 1.00	high	C
						60	3	7.67 ± 1.15	3	7.00 ± 1.00		
						120	3	12.33 ± 2.52	3	7.00 ± 1.00		
Shukla R. K. 2011 [29]	India	*Human epidermal cell line A431*, exposed for 6 h	Anatase	50	99.7	0.008	3	11.67 ± 1.20	3	9.33 ± 1.00	high	B
						0.08	3	12.67 ± 0.88	3	9.33 ± 1.00		
						0.8	3	14.67 ± 1.20	3	9.33 ± 1.00		
						8	3	15.67 ± 0.88	3	9.33 ± 1.00		
						80	3	16.00 ± 0.58	3	9.33 ± 1.00		
Srivastava R. K. 2013 [45]	India	*Human lung cancer cell line (A549)*, exposed for 24 h	Anatase	<25	NA	10	3	12.66 ± 0.33	3	5.33 ± 0.33	medium	B
						50	3	17.33 ± 0.33	3	5.33 ± 0.33		
Shukla R. K. 2013 [31]	India	*HepG2 human hepatocellular carcinoma cells*, exposed for 6 h	Anatase	30–70	99.7	1	3	8.00 ± 1.15	3	7.00 ± 0.58	high	B
						10	3	11.00 ± 1.53	3	7.00 ± 0.58		
						20	3	15.00 ± 0.58	3	7.00 ± 0.58		
						40	3	12.33 ± 0.33	3	7.00 ± 0.58		
						80	3	10.67 ± 0.88	3	7.00 ± 0.58		
Kansara K. 2015 [34]	India	*Human lung cancer cell line (A549)*, exposed for 6 h	Anatase	4–8	99.7	25	3	7.33 ± 1.20	3	6.00 ± 2.80	medium	B
						50	3	9.66 ± 2.84	3	6.00 ± 2.80		
						75	3	12.33 ± 2.96	3	6.00 ± 2.80		
						100	3	14.66 ± 2.33	3	6.00 ± 2.80		
Andreoli C. 2018 [35]	Italy	*Peripheral blood monocytes*, exposed for 24 h	Anatase	20–60	>99.5	50	2	9.0 ± 1.41	2	8.5 ± 0.71	medium	A
						100	2	10.0 ± 4.24	2	8.5 ± 0.71		
Andreoli C. 2018 [35]	Italy	*Peripheral blood monocytes*, exposed for 24 h	Rutile	30 × 100	>99.5	50	2	9.0 ± 2.83	2	7.5 ± 3.54	medium	A
						100	2	7.0 ± 2.83	2	7.5 ± 3.54		
						200	2	8.0 ± 1.41	2	7.5 ± 3.54		
Andreoli C. 2018 [35]	Italy	*Peripheral blood monocytes*, exposed for 24 h	Anatase/Rutile	45–262	>99.5	50	2	9.5 ± 0.71	2	9.5 ± 0.71	medium	A
						100	2	8.0 ± 4.24	2	9.5 ± 0.71		
						200	2	5.5 ± 2.12	2	9.5 ± 0.71		
Osman I. F. 2018 [36]	UK	*Lymphocytes from patients with respiratory diseases*, exposed for 72 h	Anatase	40–70	99.7	5	40	8.29 ± 1.55	40	8.54 ± 1.40	high	B
						10	40	11.03 ± 1.70	40	8.54 ± 1.40		
Osman I. F. 2018 [36]	UK	*Lymphocytes from healthy people*, exposed for 72 h	Anatase	40–70	99.7	5	12	4.47 ± 2.39	12	1.87 ± 1.63	high	B
						10	12	7.21 ± 1.69	12	1.87 ± 1.63		
Ünal F. 2021 [37]	Turkey	*Human lymphocytes*, exposed for 48 h	NA	<100	NA	20	3	0.30 ± 0.099	3	0.13 ± 0.066	medium	A+++
						40	3	0.30 ± 0.099	3	0.13 ± 0.066		
						60	3	0.30 ± 0.099	3	0.13 ± 0.066		
						80	3	0.17 ± 0.075	3	0.13 ± 0.066		
						100	3	0.13 ± 0.066	3	0.13 ± 0.066		
Outcomes were described as CA frequency												
Catalán J. 2011 [46]	Finland	*Human lymphocytes*, exposed for 24 h, 48 h and 72 h	Anatase	<25	99.7	6.25	2	1.25 ± 1.26(24 h) 0.50 ± 0.58(48 h) 0.25 ± 0.50(72 h)	2	0.75 ± 0.96(24 h) 0.00 ± 0.00(48 h) 0.50 ± 1.00(72 h)	high	A++
						12.5	2	0.50 ± 0.58(24 h) 0.50 ± 0.58(48 h) 1.25 ± 0.96(72 h)	2	0.75 ± 0.96(24 h) 0.00 ± 0.00(48 h) 0.50 ± 1.00(72 h)		
						25	2	0.00 ± 0.00(24 h) 0.25 ± 0.50(48 h) 0.25 ± 0.50(72 h)	2	0.75 ± 0.96(24 h) 0.00 ± 0.00(48 h) 0.50 ± 1.00(72 h)		
						50	2	0.50 ± 0.58(24 h) 0.25 ± 0.50(48 h) 0.50 ± 1.00(72 h)	2	0.75 ± 0.96(24 h) 0.00 ± 0.00(48 h) 0.50 ± 1.00(72 h)		
						100	2	0.00 ± 0.00(24 h) 1.00 ± 0.82(48 h) 0.75 ± 0.96(72 h)	2	0.75 ± 0.96(24 h) 0.00 ± 0.00(48 h) 0.50 ± 1.00(72 h)		
						150	2	0.25 ± 0.50(24 h) 1.25 ± 0.50(48 h) 0.50 ± 0.58(72 h)	2	0.75 ± 0.96(24 h) 0.00 ± 0.00(48 h) 0.50 ± 1.00(72 h)		
						300	2	1.00 ± 1.15(24 h) 1.00 ± 0.82(48 h) 0.50 ± 0.58(72 h)	2	0.75 ± 0.96(24 h) 0.00 ± 0.00(48 h) 0.50 ± 1.00(72 h)		
Ünal F. 2021 [37]	Turkey	*Human lymphocytes*, exposed for 24 h, 48 h	NA	<100	NA	20	3	6.00 ± 1.37(24 h) 5.33 ± 1.30(48 h)	3	1.33 ± 0.66(24 h) 1.33 ± 0.66(48 h)	medium	A+++
						40	3	6.67 ± 1.44(24 h) 3.00 ± 0.98(48 h)	3	1.33 ± 0.66(24 h) 1.33 ± 0.66(48 h)		
						60	3	4.33 ± 1.17(24 h) 3.33 ± 1.03(48 h)	3	1.33 ± 0.66(24 h) 1.33 ± 0.66(48 h)		
						80	3	5.00 ± 1.26(24 h) 3.33 ± 1.03(48 h)	3	1.33 ± 0.66(24 h) 1.33 ± 0.66(48 h)		
						100	3	6.00 ± 1.37(24 h) 4.00 ± 1.13(48 h)	3	1.33 ± 0.66(24 h) 1.33 ± 0.66(48 h)		

^1^ NA: not applicable; *n*: sample size; SD: standard deviation; T DNA%: the percentage of DNA in tail; TL: tail length; OTM: olive tail moment; MF: mutation frequency; BiMN: no. of micronucleus/1000 binucleated cells; CA frequency: percentage of cells exhibiting chromosomal aberrations.

## Data Availability

The research data can be found in the figures and tables within this article.

## Abbreviations

TiO_2_ NPs	Nanoparticles Titanium Dioxide Nanoparticles
EFSA	European Food Safety Authority
WoS	Web of Science
CNKI	China National Knowledge Infrastructure
T DNA%	the percentage of DNA in tail
TL	tail length
OTM	olive tail moment
MF	mutation frequency
MN	micronucleus
CA	chromosomal aberrations
TRAM	toxicological data reliability assessment method
CDC	Center for Disease Control and Prevention
SMD	standardized mean difference
CI	confidence interval
ROS	reactive oxygen species
DDR	DNA damage response
